# Ecological correlates of Himalayan musk deer *Moschus leucogaster*

**DOI:** 10.1002/ece3.4435

**Published:** 2018-11-24

**Authors:** Paras Bikram Singh, Pradip Saud, Douglas Cram, Kumar Mainali, Arjun Thapa, Nar Bahadur Chhetri, Laxman Prasad Poudyal, Hem Sagar Baral, Zhigang Jiang

**Affiliations:** ^1^ Key Laboratory of Animal Ecology and Conservation Biology Institute of Zoology Chinese Academy of Sciences Beijing China; ^2^ University of Chinese Academy of Science Beijing China; ^3^ National Trust for Nature Conservation Khumaltar Nepal; ^4^ Extension Animal Sciences and Natural Resources New Mexico State University Las Cruces New Mexico USA; ^5^ Department of Biology University of Maryland College Park Maryland USA; ^6^ Institute of Forestry Tribhuvan University Pokhara Nepal; ^7^ Department of National Parks and Wildlife Conservation Kathmandu Nepal; ^8^ Zoological Society of London, Nepal Office Kathmandu Nepal; ^9^ School of Environmental Sciences Charles Sturt University Albury New South Wales Australia

**Keywords:** crown cover, elusive species, latrines, shrub diversity

## Abstract

Himalayan musk deer (*Moschus leucogaster*; hereafter musk deer) are endangered as a result of poaching and habitat loss. The species is nocturnal, crepuscular, and elusive, making direct observation of habitat use and behavior difficult. However, musk deer establish and repeatedly use the same latrines for defecation. To quantify musk deer habitat correlates, we used observational spatial data based on presence–absence of musk deer latrines, as well as a range of fine spatial‐scale ecological covariates. To determine presence–absence of musk deer, we exhaustively searched randomly selected forest trails using a 20‐m belt transect in different study sites within the Neshyang Valley in the Annapurna Conservation Area. In a subsequent way, study sites were classified as habitat or nonhabitat for musk deer. A total of 252 plots, 20 × 20 m, were systematically established every 100 m along 51 transects (each ~0.5 km long) laid out at different elevations to record a range of ecological habitat variables. We used mixed‐effect models and principal component analysis to characterize relationships between deer presence–absence data and habitat variables. We confirmed musk deer use latrines in forests located at higher elevations (3,200–4,200 m) throughout multiple seasons and years. Himalayan birch (*Betula utilis*) dominated forest, mixed Himalayan fir (*Abies spectabilis*), and birch forest were preferred over pure Himalayan fir and blue pine (*Pinus wallichiana*) forest. Greater crown cover and shrub diversity were associated with the presence of musk deer whereas tree height, diameter, and diversity were weakly correlated. Topographical attributes including aspect, elevation, distance to water source, and slope were also discriminated by musk deer. Over‐ and understory forest management can be used to protect forests likely to have musk deer as predicted by the models to ensure long‐term conservation of this rare deer.

## 1 INTRODUCTION

Musk deer (*Moschus* spp.) comprise seven species and exist in thirteen countries in Asia (DNPWC, 2016; Wilson & Russell, 2011; Zhou, Meng, Feng, & Yang, 2004). Each species has its own restricted range. For example, Anhui musk deer (*Moschus anhuiensis*) is found in Anhui Province, China (Su, Wang, & Wang, 2000) and Kashmir musk deer (*Moschus cupreus*) is found from Kashmir, India to Pakistan and eastern Afghanistan (Ostrowski, Rahmani, Ali, Ali, & Zahler, 2016). Himalayan musk deer (*Moschus leucogaster*; hereafter musk deer) is found on the southern slopes of the Himalayas in Bhutan, India, Nepal, and marginally in China (Timmins & Duckworth, 2015; Wilson & Russell, 2011). Worldwide, populations of musk deer have dramatically dwindled to half of the original size in three generations (approximately 21 years) primarily because of poaching and habitat degradation (Green, 1986; Homes, 2004; Timmins & Duckworth, 2015). Musk deer are poached to acquire their musk pods (found only in males), which have been traded for traditional medicines and perfumes in China, India, and other countries since the 5th century (Feng, You, Yong, Li, & Gu, 1981; Jiang, Meng, & Wang, 2002). Despite the fact that poaching targets males, snares indiscriminately kill females and juveniles as well (Sheng & Liu, 2007; Sheng & Ohtaishi, 1993; Yang, Meng, Xia, & Feng, 2003). The global demand for musk pods is pushing musk deer toward extinction (Homes, 2004; Yang et al., 2003). Therefore, musk deer have been categorized as endangered (Timmins & Duckworth, 2015) and are listed in Appendix I in the Convention on International Trade in Endangered Species of Wild Fauna and Flora (CITES, 2015). In Nepal, three species of musk deer (i.e., Himalayan, alpine [*Moschus chrysogaster*], and black [*Moschus fuscus*]) are strictly protected under the National Park and Wildlife Conservation Act (Baral & Shah, 2008; Jnawali et al., 2011).

In addition to poaching, the other prevailing threats to musk deer are habitat destruction and degradation (Green, 1986; Ilyas, 2014; Yang et al., 2003). Habitats in the Himalayas are being threatened by anthropogenic pressures such as intensive livestock grazing, fuel wood cutting, fodder collection, establishment of hydropower plants, and road development (Dorji, Vernes, & Rajaratnam, 2011; Grumbine & Pandit, 2013; Thapa, Hu, & Wei, 2018; Vinod & Sathyakumar, 1999). As a result, suitable habitat for musk deer is mainly confined to protected areas with fragmented habitat between reserves. The Himalayas and associated environments are also vulnerable to climate change (Beaumont et al., 2011; Xu et al., 2009).

Musk deer are solitary and shy forest dwellers with crepuscular and nocturnal activity patterns (Green, 1985; Kattel, 1993). These behaviors in association with their densely vegetated habitat result in the species being elusive to predators (e.g., common leopard (*Panthera pardus),* snow leopard (*Uncia uncia)*, and golden jackal (*Canis aureus*)) and humans. However, musk deer establish latrines by defecating repeatedly at the same location within their home range (0.15 to 0.31 km^2^; Green, 1985; Kattel, 1993). Such behavior is believed to facilitate olfactory/chemical communication among individuals (Green, 1987b; Meng, Cody, Gong, & Xiang, 2012b; Meng, Li, & Meng, 2012a). In many mammals, including musk deer, latrines are used for territorial defense by establishing latrines in the periphery and core areas of the habitat (Grau & Walther, 1976; Green, 1985, 1987b; Mykytowycz, Hesterman, Gambale, & Dudziński, 1976; Wronski, Apio, & Plath, 2006). Because latrines are confirmed evidence of deer presence in a particular habitat, the ecological and environmental covariates of latrine locations can provide insights into preferred habitats and conditions throughout the landscape. This knowledge helps formulate effective conservation planning and habitat management. Although it has been long known that musk deer inhabit the Himalaya range in mature conifer and broadleaved forests (Green, 1986; Kattel, 1993; Sathyakumar, 1992), fine scale ecological correlates of its habitat have not been studied previously. In this study, we analyzed habitat correlates of musk deer in the Annapurna region of central Himalaya. We hypothesized that the presence of latrines in a particular habitat type is linked with biotic and abiotic characteristics such as crown cover, tree height and diameter, shrub diversity, and elevation.

## 2 MATERIALS AND METHODS

### 2.1 Study area

**Figure 1:**
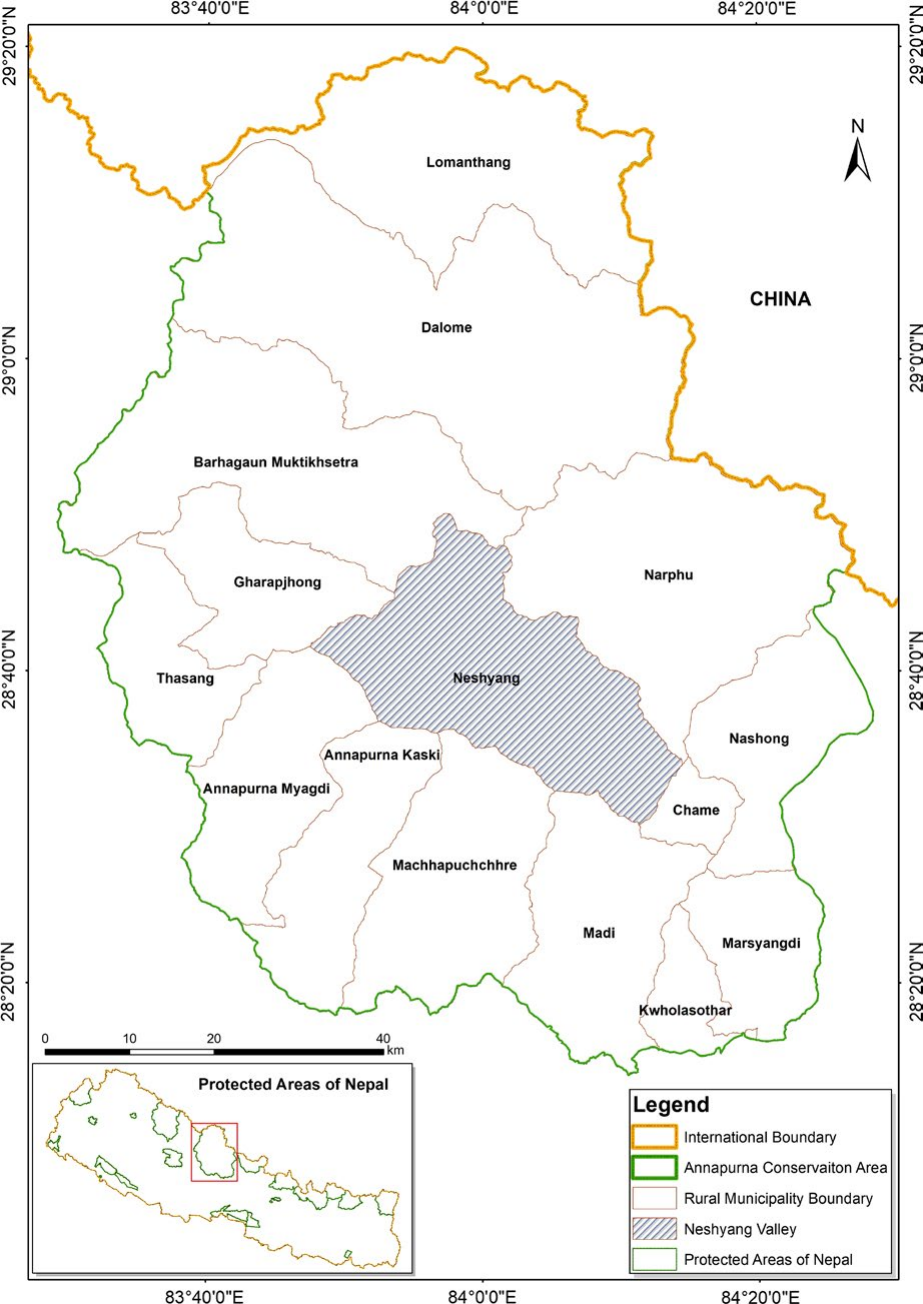
Neshyang valley, Manang, Annapurna Conservation Area

The Annapurna Conservation Area (ACA), located in the western region of Nepal (28°13′ 48″ to 29°19′ 48″N and 83°28′ 48″ to 84°26′ 24″E), covers 7629 km^2^ making it the largest protected area in the country (DNPWC, 2016, NTNC, 2015). It comprises a wide range of habitats from subtropical forest to alpine tundra. It harbors 105 species of mammals, 488 species of birds, 20 species of fishes, 23 species of amphibians, 40 species of reptiles and 347 species of butterflies (DNPWC, 2016). Musk deer are generally found in the river valleys of Manang, Mustang and the southern slopes of the Annapurna Range. Among these sites, Neshyang Valley, in Manang (690 km^2^; part of the ACA) is prime habitat for musk deer (Figure 1). Therefore, we selected Neshyang Valley as our study site. The valley consists of four vegetation types: blue pine (*Pinus wallichiana*) forest (hereafter pine forest), Himalayan fir (*Abies spectabilis*) forest (hereafter fir forest), Himalayan birch (*Betula utilis*) forest (hereafter birch forest) and mixed forest. Mixed forests are comprised of either Himalayan birch and fir or blue pine and Himalayan fir or all three species of trees.

ACA has been managing by local people since 1985 (Baral, Stern, & Heinen, 2010) under the popular and effective theme “local community participation in nature conservation” (Khadka & Nepal, 2010; Kubo & Supriyanto, 2010; Twyman, 2000). The approach of integrated conservation and development (ICDP) has been effective in conservation of flora and fauna. This approach has been executing by forming 969 institutions of local people (Bajracharya, Gurung, & Basnet, 2007). On the other hand, 147 staffs of ACA including conservation officers and rangers have been deploying to strengthen local institutes under national parks and wildlife conservation act 1973 to save nature. Under this program, commercial logging and hunting are strictly prohibited, but local people can collect dead trees for fuelwood. Local people in the community are also allowed to harvest some trees for building cottages. However, local people must get permission from conservation area management committee and office of ACA. It is important that, the local people specially in Neshyang and Mustang valleys of ACA where musk deer are abundant believe on Buddhism and they do not kill animals (Gurung & Thapa, 2004). Hence, only the people from outside of the valley were apprehended by the office of ACA involving in poaching of musk deer and snow leopard. Due to the local scenario and participatory conservation, all kinds of habitat and animals are protected strictly. Furthermore, rotational grazing of agricultural fields after harvest, selected forest area and pasture land above tree line have been effective in conserving wildlife habitat of this area (Bajracharya, Furley, & Newton, 2005).

### 2.2 Preliminary survey

Neshyang Valley was divided into four blocks: Pisang‐Ghyaru, Humde‐Quebesi, Bhraka‐Manang and Khangsar‐Gunsang based on the administrative boundaries of the district. Within each block, forested areas separated by natural features such as river, streams, ridges and gullies were selected as study sites. A total of eleven study sites were randomly selected (five sites in Pisang‐Ghyaru, two sites in Humde‐Quebesi, two sites in Bhraka‐Manang and two sites in Khangsar‐Gunsang). A preliminary visit of each site was made to determine whether musk deer latrine signs were present or absent from those sites. For this purpose, a forest trail running from lower to higher elevation was randomly selected. Forest trails are defined as natural paths created inside the forest by livestock, wildlife and humans. Approximately 20 m of belt transect was selected in forest in such way that 10 m–10 m on each side of the trail in each site was exhaustively searched between late April and early May 2016 for latrines along slope until the forest ended. Search teams, under the direction of the lead author, consisted of rangers, members of conservation area management committee and forest guards. Based on exhaustive searches and consultation with local inhabitants and ACA staff, when we did not retrieve latrine signs or deer information in a site then we acknowledged those sites as “nonhabitat sites” otherwise “habitat sites.” Hereafter, we designated four sites “nonhabitat sites” (Pisang lake, Lower Ghyaru, Munje Quebesi, and Tare Gomba) and seven sites “habitat sites” (Dhikurpokhari, Pisang, Ghyaru, Humde, Bhraka Manang, Khangsar, and Gunsang) for musk deer.

### 2.3 Sampling design and data collection

**Figure 2:**
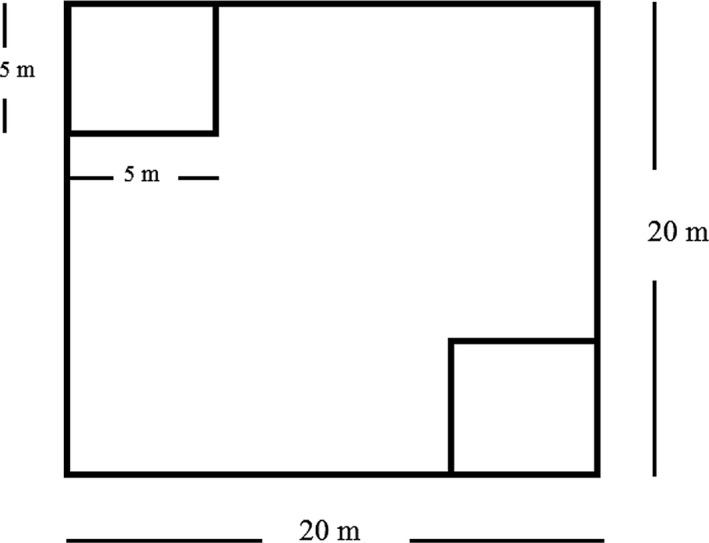
Layout of quadrate plots and nested plots for biophysical sampling

After classifying study sites as habitat and nonhabitat, we visited all sites between May–July 2016 to collect the data on latrine presence/absence and characterize overstory vegetation composition and structure using line transects. Each study site was divided into 1 × 1 km grid and the grids were randomly selected. Forest types within the grid were divided into pine, fir, birch and mixed forest on the basis of tree species. These forest types range between 2,900–3,600 m, 3,000–3,800 m, 3,800–4,200 m and 3,600–4,000 m, respectively. In regard to mixed forests, three types were noted in quadrat plots based on dominant species: birch mixed (BirMixed) where birch was the dominant species; fir mixed (FirMixed) where fir was the dominant species and pine mixed (PineMixed) where pine was the dominant species. A total of 51 transects, each 0.5 km in length was established at different elevations from 2,900 to 4,200 m. On each line transect running at different elevations from lower to higher elevations, five quadrats were placed at 100 m intervals and the geographical location of each quadrat was recorded. A total of 144 and 108 quadrat plots were sampled in habitat and nonhabitat sites respectively. Vegetation data and biophysical characteristics were recorded in each quadrat as well as presence and absence of latrine in each quadrat were also noted (Table 1). We also recorded the number of shrub species as shrub diversity by placing two 5 × 5 m nested plots in opposite corners of each quadrat (Figure 2).

**Table 1:** Variables measured in quadrate of 20 × 20 m and nested plot of 5 × 5 m

Measured variables	Unit	Equipment
Geographical locations	UTM	GPS
Altitude above sea level	Meters (m)	Altimeter
Aspect	N, S, E,W, NE, SE, NW, SW	Compass
Slope	Degree	Santo Clinometer
Dominant Height (Dmht)	Meters (m)	Santo Clinometer
Diameter at Breast Height: greater than 30 cm (Dbhg30)	Stems per hectare (stems/ha)	D‐tape
Diameter at Breast Height: greater 10 cm ≤ 30 cm (Dbhg10)	Stems/ha	D‐tape
Diameter at Breast Height: less than and equal to 10 cm (Dbh10)	Stems/ha	D‐tape
Tree per hectare (TPH)	Stems/ha	Direct count
Shrub diversity (Shrubdiv)	Number of shrub species per hectare	Direct count
Tree diversity (Treediv)	Number of tree species per hectare	Direct count
Crown Cover in Percentage (CCPCT)	Percentage (%)	Crown densitometer
Distance from water (DisWater)	Meters (m)	GPS
Latrine status	Fresh, Old, Fresh & Old, Very Old	Direct observation and smelling
Latrine location	Under tree/under canopy/space under the rock	Direct observation

Musk deer latrines found in each quadrat were categorized into four groups: fresh; (defined as moist and pungent pellets), old; (defined as shiny pellets with no discoloration and no cracks), fresh and old; (combination of previous two categories), or very old; (discolored, dried and cracked). We visited all sites again between May–July 2017 and searched the same quadrats to collect data on musk latrine presence/absence. This time period of a year was selected partly because snow in the forest had melted completely (Polunin & Stainton, 1984; Raskoti & Ale, 2013). In addition, musk deer do cover fresh pellets in the latrine by shaded leaves, soil and old pellets and use some specific latrines during mating season (October to November) whereas they do not cover fresh latrines and use many latrine sites from April to August (Green, 1985).

### 2.4 Data analysis

Vegetation structure and topographical attributes with reference to latrine presence and absence in the habitat sites were analyzed separately from nonhabitat sites. Moreover, the similar types of data as habitat sites collected from nonhabitat sites were compared with habitat sites to understand the habitat discrimination of musk deer in the study area.

#### 2.4.1 Latrine count and distribution

Musk deer latrine count data from 2016 (hereafter YR16) and 2017 (hereafter YR17) were analyzed using contingency tables. Their distribution along topographical aspect, forest type and location were shown using bar plots. Mean musk deer latrine counts between years were compared. For simplicity, latrine count categories were marked as “0,” “1,” and “2+” (i.e., two or greater). From habitat sites, 144 plots were sampled in 2016 with 42 plots having “1” latrine and 48 plots having “2+” latrines. Revisited plots in 2017 had 41 plots with “1” latrine and 48 plots with “2+” latrines. Chi‐square (*χ* ^2^) was used to test whether latrine counts were independent of year and the difference in latrine proportion. Musk deer latrine counts were converted into a binary response of zero and one, where “0” represented no latrines (absence) and “1” represented at least one latrine per sample plot (presence). Hereafter, latrine presence–absence refers to a binary response. McNemar's test was used for matched binary data to determine differences in latrine presence–absence between years (Agresti, 2007). The above‐mentioned test statistics were also used to evaluate average latrine count distribution along aspect, forest type and location. Whenever *χ* ^2^ test suggested significant latrine associations, residuals were used to show correlation between latrine counts and habitat variables (Agresti, 2007).

#### 2.4.2 Habitat attributes and latrine correlation

The least mean difference in habitat variables between latrine presence and absence from habitat site was evaluated using the mixed‐effects modeling approach. In wildlife studies, this modeling approach emphasizes the random effect on population dynamics in space and time and also allows extrapolation to individuals and populations beyond the study sample (Bolker et al., 2009). The model structure where transects were nested within site was used as random effect because plots sampled within each transect were assumed to be correlated. This model structure allowed the intercept to vary with transect within site. The mixed‐effects model was executed using Proc Mixed in SAS Enterprise Guide 7.1 (SAS, 2014) with Kenward‐Rodgers approximation used to determine significance. Mean difference in habitat variables at *α* = 0.05 between latrine presence and absence were reported but random effects were not reported. Correlation of average latrine count was established with habitat variables using Kendall tau (*τ*) rank correlation (Kloke & McKean, 2014). Habitat variables indicating significant correlation with latrine counts were also plotted.

#### 2.4.3 Modeling latrine presence–absence

Latrine presence–absence was modeled as a binary distribution using logit function in multilevel mixed‐effect model with “glmer” function using “lme4” package (Bates, Maechler, Bolker, & Walker, 2015) in R (R Core Team, 2016). We fitted the full model with all potential candidate variables and backward stepwise selection method was used to select the model with the best covariates having the smallest Akaike Information Criteria (AIC) value. Later, the selected covariates were fitted using mixed‐effects model where the model structure used two random effects—one associated with site level and another associated with transect nested within site as multiple transects were sampled within a site—because we were not interested in the statistical significance of these terms. We also compared the selected model with the null model (without covariates) fitted as multilevel mixed model using likelihood ratio test (Zuur, Ieno, Walker, Saveliev, & Smith, 2009).

Performance of the best model was evaluated using a receiver‐operating characteristics (ROC) curve as compared to the null model using “pROC” package (Robin et al., 2011) in R. The ROC plot provides area under the curve as a useful indicator of model performance in evaluating presence–absence in ecology (Manel, Williams, & Ormerod, 2001). The significance of each variable in the model predicting latrine presence–absence was also evaluated by plotting predicted values (probability) against response (latrine presence–absence) based on model fit.

#### 2.4.4 Habitat versus nonhabitat

##### Comparing habitat covariates

The least mean difference in habitat variables between habitat and nonhabitat site was evaluated using the multilevel mixed‐effects modeling approach, where the random effect was associated with sites and with transect nested within site transects. Mean difference in habitat covariates between habitat and nonhabitat site was reported at *α* = 0.05, the significance level, but random effects were not reported.

##### Multivariate analysis

To examine factors contributing to musk deer habitat versus nonhabitat sites, we used principal component analysis (PCA) in Canoco 5 (Ter Braak & Šmilauer, 2012). PCA was run on the pooled data between habitat site and nonhabitat site with ten habitat attributes variables. Sample response traits were (a) habitat and nonhabitat sites and (b) latrine presence–absence samples. This analysis is a form of PCA with supplementary variables or simply unconstrained analysis with supplementary variables (Šmilauer & Lepš, 2014; Ter Braak & Šmilauer, 2012). The PCA analysis was performed in centered and standardized response.

## 3 RESULTS

### 3.1 Musk latrine count and status

**Figure 3:**
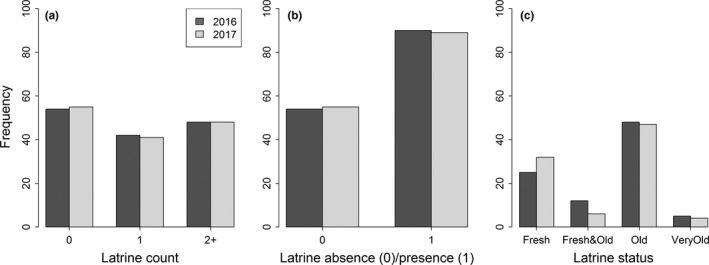
Frequency of musk latrine based on latrine count (a), latrine absence and presence (b), and latrine status (c)

A total of 144 plots were sampled with an average of 90 plots indicating musk latrine use in potential habitat of musk deer. The average proportion of latrine presence was 0.62 and latrine absence was 0.38. The observed categories of latrine count were independent of the year (*χ* ^2^ = 0.021, *p* = 0.99), indicating that observing the frequency of musk latrine count categories (0, 1 and 2+) was not different across years (Figure 3a). However, during a given year, the frequencies of the three categories were significantly different between at least one pair of the groups (*χ* ^2^ = 8.804, *p* = 0.012). Similar to that, the frequencies of samples with presence or absence of latrines was also independent of years (Figure 3b), indicating that observed latrine presence and latrine absence sites were not different in the two studied years. But the exact McNemar test for matched binary data showed that the proportion of latrine presence sites were significantly different than the proportion of latrine absence sites during YR16 and YR17 (*p* = 0.004).

Fisher's exact test indicated that counts of musk latrine status (Figure 3c) were also independent of the year (*p* = 0.41). But the average observed proportion of old latrines (53.2%) and fresh latrines (31.4%) were significantly higher (*p* < 0.01) than both fresh and old (10.3%) and very old (5.1%) latrine. Although increased Fresh latrine count and decreased Fresh & Old latrine count was observed in the second year (Figure 3c), the latrine count was not significantly different between years for both Fresh (*p* = 0.23) and Fresh & Old latrine (*p* = 0.14) latrine status.

### 3.2 Latrine distribution by aspect, forest type, elevation, and location

As the musk deer latrine count was not significantly different between years and distribution was almost identical, average musk latrine count was used to show distribution with aspect, elevation, forest type and location.

#### 3.2.1 Aspect

**Figure 4:**
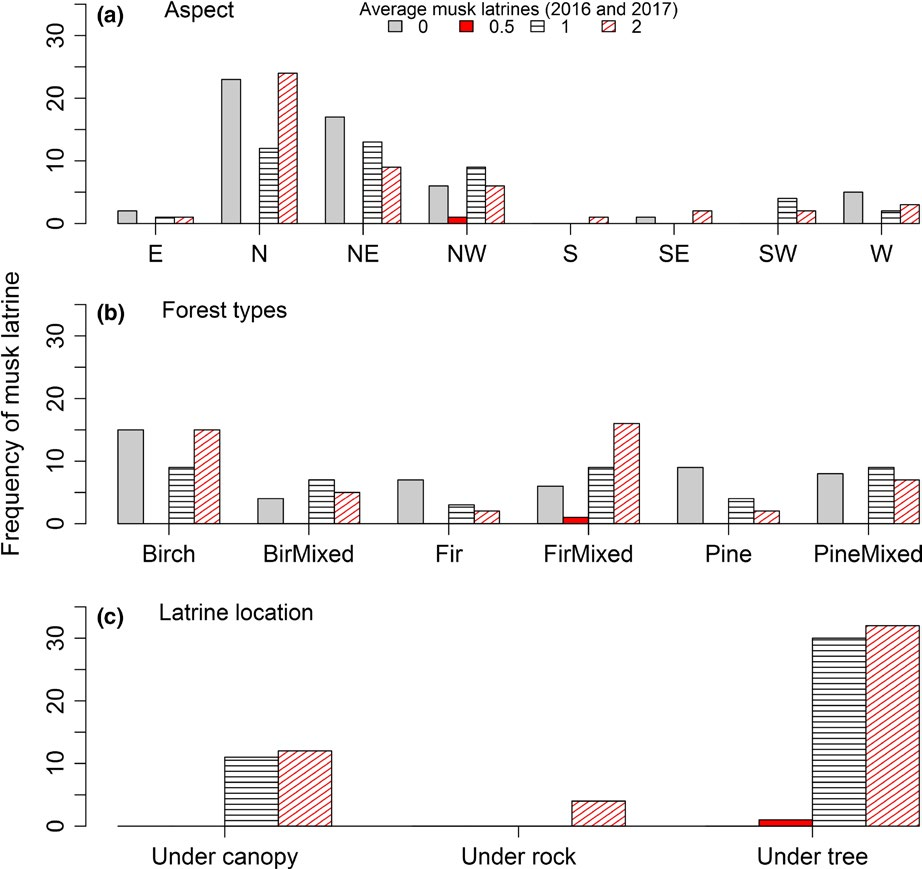
Average frequency of musk latrine based on topographical aspect (a), forest types (b), and latrine location (c)

The distribution of the latrine count category based on topographical aspect is shown in Figure 4a. The largest fraction of latrine presence was observed on the N aspect (40.2%) and followed by the NE aspect (24.6%), NW aspect (17.3%), SW (6.7%), W (5.6%), SE (2.2%), E (2.2%) and S (1.1%). When the slopes were clumped for just North versus South, the vast majority (82.1%) of latrine presences were found on northward‐facing slopes. This compares to 10% latrine presences on the southward facing slopes. The latrine absence count was high on the N aspect (42.2%), followed by NE (31.2%), NW (11.9%), W (9.2%), E (3.7%) and SE (1.8%). However, S and SW aspects had no latrine absence records. In a collective way, northward facings slopes had 85% of absences, compared to 1.8% in all the southward facing slopes. Statistical analysis was not conducted for those aspects contributing less than 5% in their total latrine count. Although, high frequency of latrine count response was observed on N, NE and NW aspect, no significant difference in the proportion of latrine count was observed among aspects for zero latrine (*χ* ^2^ = 3.15, *df* = 3, *p* = 0.364), one latrine (*χ* ^2^ = 3.62, *df* = 3, *p* = 0.164) and 2+ ‐latrine category (*χ* ^2^ = 2.02, *df* = 3, *p* = 0.206).

#### 3.2.2 Forest type and elevation

Among forest types, the average high proportion of total musk latrines observed by count category were in FirMixed (18.7%) followed by birch forest type (16.9%), PineMixed (11.3%), BirMixed (8.9%), pine (4.4%) and fir (3.7%). The average high proportion of zero latrine count was reported in Birch forest (11%) followed by Pine (6.6%), PineMixed (5.9%), Fir (5.1%), FirMixed (4.4%) and BirMixed (2.9%; Figure 4b). The observed frequencies of latrine count category were dependent on forest types (*χ* ^2^ = 24.872, *df* = 5, *p* < 0.001). Standardized residuals ([observed count–expected count)]/√expected count) greater than positive two indicated strong positive association of latrine counts with Birch and FirMixed forest. Residuals with less than negative two showed strong negative associations with fir and pine forests. Latrines were recorded from the altitudinal range 3,200 m to 4,200 m in various types of forest, but latrine presence samples were highly concentrated between 3,650 m and 4,000 m.

#### 3.2.3 Latrine location

Locations of musk deer latrines were identified as under tree, under canopy and space under rock (space at the base of boulder, cliff and under overhanging rock; see Table 1). In both years, a higher frequency of latrines was observed under tree than under canopy with a small number inside space under rock (Figure 4c). On average, 69.0% of total musk latrines were observed under tree, while 26.4% were observed under canopy and the remaining 4.6% were observed under rock. Although observed latrine count proportion was significantly higher under tree than under canopy (*χ* ^2^ = 29.85, *p* < 0.001), the observed latrine counts category were independent of the latrine location (*χ* ^2^ = 3.41, *p* = 0.233).

### 3.3 Habitat attributes and latrine correlation

Difference of least square means for habitat variables between presence and absence latrine plots using mixed‐effect models (Table 2) indicated that mean values of Crown Cover Percentage (CCPCT), Dominant height (Dmht), shrub diversity and tree diversity were significantly greater on latrine presence plots than latrine absence plots. Other habitat variables such as tree density (trees per hectare) of greater than 30 cm in diameter at breast height (Dbhg30) and total tree density (TPH) were also significantly different between latrine presence and absence plots (Table 2). Topographical attributes such as elevation, slope, and distance to water were not statistically different between latrine presence and absence plots.

**Table 2:** Comparison of means ± standard error for all measured variables from latrine presence and absence plots using mixed‐effect model, where the random effect of a transect was nested within site, and nonparametric correlation (Kendal tau, *τ*) between average latrine counts with habitat variables

Variables	Least square means			Correlation	
	Presence	Absence	*p*‐Value	*τ*	*p*‐Value
Elevation (m)	3,774 ± 42	3,784 ± 43	0.331	0.04	0.517
CCPCT (%)	75.8 ± 2.8	63.7 ± 3.3	0.001	0.09	0.145
Dbhg30 (stems/ha)	25 ± 4	14 ± 5	0.027	0.08	0.252
Dbhg10 (stems/ha)	199 ± 24	169 ± 26	0.139	0.19	0.004^a^
Dbh10 (stems/ha)	263 ± 37	178 ± 43	0.061	0.15	0.020^a^
Distance to water (m)	342 ± 34	331 ± 37	0.706	0.03	0.622
Dmht	7.3 ± 0.3	5.9 ± 0.4	<0.001	0.15	0.019^a^
Shrub diversity (species/plot)	2.8 ± 0.1	2.1 ± 0.2	<0.001	0.26	0.001^a^
Slope (degree)	27.2 ± 1.5	27.9 ± 1.7	0.648	0.01	0.861
TPH (stems/ha)	487 ± 50	363 ± 56	0.025	0.17	0.009^a^
Tree diversity (species/plot)	1.7 ± 0.1	1.3 ± 0.1	0.001	0.22	0.003^a^

*Notes*

CCPCT: Crown cover in percentage; Dbhg30: density of diameter at breast height greater than 30 cm; Dbhg10: density of diameter at breast height between >10 and ≤30 cm; Dbh10: density of diameter at breast height between ≤10 cm; Dmht: mean dominant height (m); TPH: total tree density.

a Significance at *α* = 0.05 level.

We used Kendall tau (*τ*) rank correlation, a nonparametric test for determining dependence. This indicated a significant positive correlation between latrine count and the following covariates: tree density, tree diversity and shrub diversity (Table 2). Correlation was weak for observed average musk latrine counts with covariates, but it was better for shrub diversity (*τ* = 0.26) and tree diversity (*τ* = 0.22).

**Figure 5:**
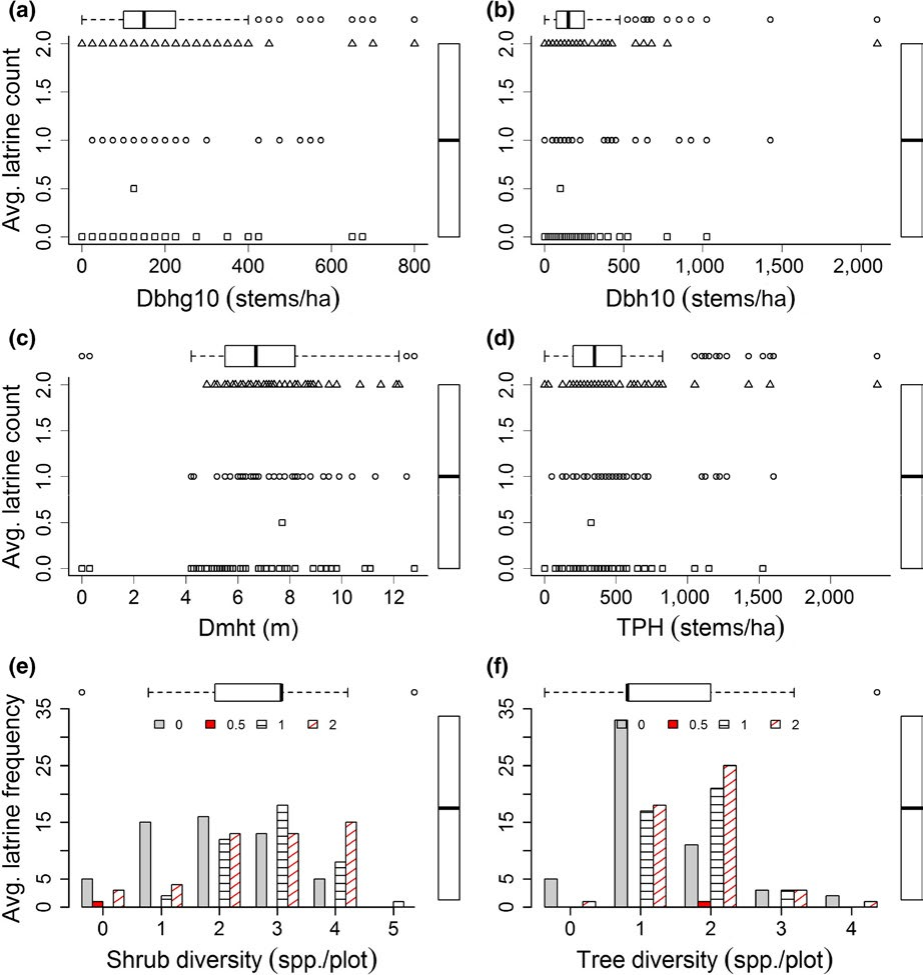
Distribution of latrine counts versus the significantly correlated variables. Scatter plot with side‐by‐side box plot shows latrine count distribution against for Dbhg10 (a), Dbh10 (b), Dmht (c), and TPH (d). Bar plot with side‐by‐side box plot shows latrine count distribution against shrub diversity (e) and tree diversity (f)

Based on the above correlation analysis (Table 2), the distribution of average latrine count against variables having significant p‐value is shown in Figure 5 using scatter and bar plots along with side‐by‐side box plots. Median latrine count was one latrine for all variables (Figure 5). In addition, for one latrine count the median stem density was 150 stems/ha for tree diameter greater than 10 cm (Dbhg10). The median value was 150 stems/ha for tree diameter less than and equal to 10 cm (Dbh10), 6.7 m for Dmht, 3 stems/ha for shrub species diversity, 350 stems/ha for TPH and one species as tree diversity (Figure 5).

### 3.4 Modeling latrine presence–absence and effect of habitat variables

Modeling latrine presence and absence as a binary response with generalized linear mixed‐effects result showed that habitat variables such as CCPCT, Dmht, shrub diversity and tree diversity had a significant effect on the probability of musk latrine presence (Table 3). The effect of habitat variables on musk latrine observation probability also varied due to significant random effects of sites and transects within site. The selected mixed‐effects model was significantly different from the null model (without covariates) fitted with mixed‐effects approach in likelihood ratio test (*χ* ^2^ = 57.6; *p* < 0.001). The model indicated that variability due to random effects in predicting latrine presence from transects within site was large than from sites.

**Table 3:** Parameter estimates and standard errors of the mixed‐effects model to predict musk latrine presence and absence based on latrine sites in Neshyang Valley, Manang, Nepal

Effects	Estimate	Standard error	*p*‐Value
Fixed			
Intercept	−4.93171	1.11225	<0.0001
CCPCT	0.02195	0.01025	0.03217
Dmht	0.28459	0.10564	0.00706
Shrub diversity	0.49403	0.15676	0.00162
Tree diversity	0.59085	0.29146	0.04264
Random			
Sites (*σ*_s_^2^)	0.6146		
Transect within sites (*σ*_ts_^2^)	0.8676		
AIC	324.3		

*Note*

CCPCT: Crown cover in percentage; Dmht: mean dominant height.

**Figure 6:**
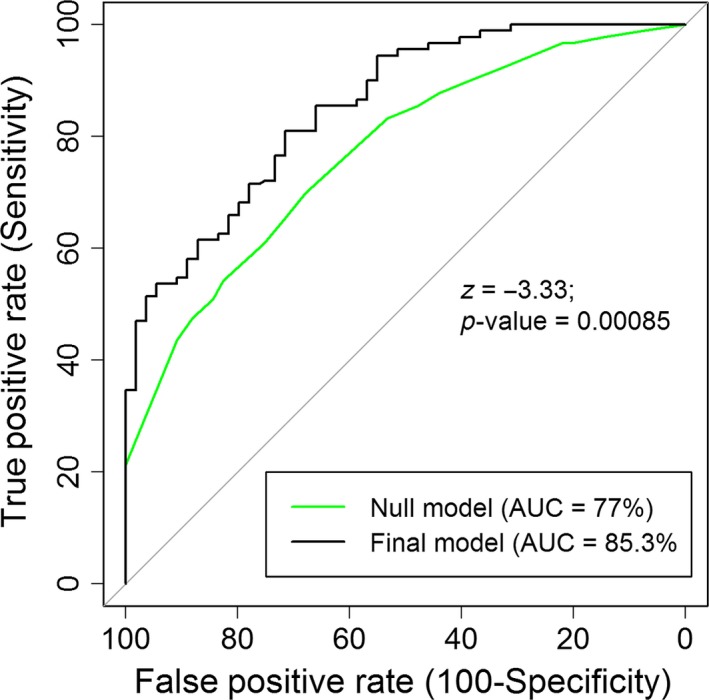
Comparing receiver‐operating characteristic (ROC) curves between null and final model. The area under the curve (AUC) of final model was significantly higher than the null model fitted as multilevel mixed‐effects model. The random variance component of the null model provided the power to make 77% correction

DeLong's test for the paired ROC curves showed that the area under the curve (AUC) of the selected model was (85.3%) significantly larger (*z* = −3.33, *p*‐value = <0.001) than the null model (77%; Figure 6); therefore, the selected covariates were highly appropriate to predict latrine presence and absence. Analysis of the ROC curves returning the point with the sum of sensitivity and specificity showed that the best model had a discrimination threshold of 0.57 to classify binary response as latrine presence and absence because this threshold minimized the classification errors of the model. The true positive rate (sensitivity) was 81% and true negative rate (predicting actual latrine absence) was 69.7%, which indicated that the model predicted a better number of actual latrine presence samples than actual latrine absence samples.

**Figure 7:**
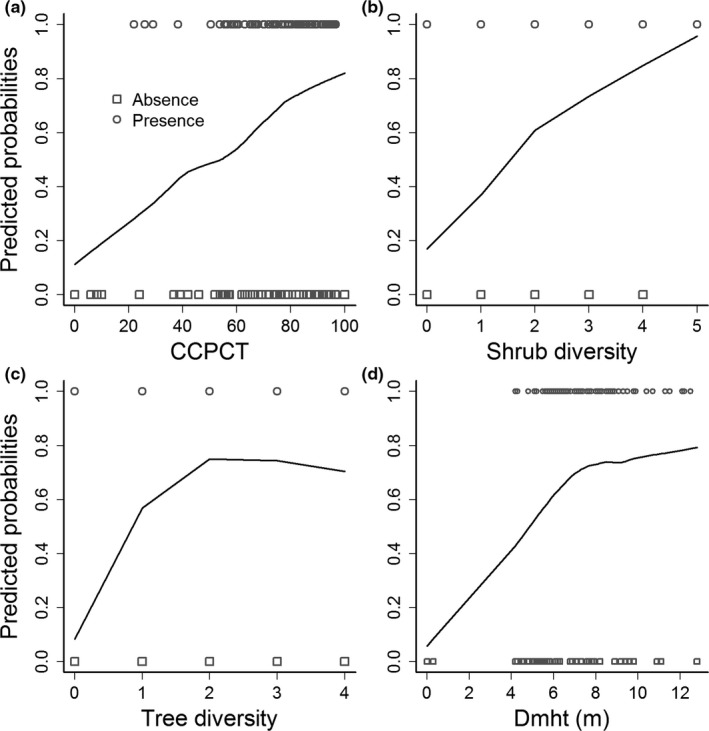
Predicted values from model fit against response (latrine absence and presence), for particular model term conditioning on random effects of mixed‐effects modeling

Based on model estimates, the predicted probability of latrine presence and absence against habitat variables is shown in Figure 7. Shrub diversity exhibited higher accuracy in discriminating presence and absence sites of latrine than other covariates. Variables such as CCPCT and shrub diversity showed an almost a linear trend in predicted probabilities of latrine presence (Figure 7a,b), whereas tree diversity and Dmht exhibited an asymptotic relationship (Figure 7c,d). As tree diversity becomes greater than two and mean Dmht greater than 7 m, these variables do not show additional effects on predicting latrine presence. The point where the predicted probabilities curve becomes flat is the optimum tree diversity and Dmht for predicting musk latrine presence (Figure 7c,d).

### 3.5 Comparing habitat versus nonhabitat sites

#### 3.5.1 Difference in habitat covariates

We did not detect any latrines in 108 sampled plots on 21 transects from four sites adjacent to occupied sites within Neshyang Valley. The least square mean comparison based on multilevel mixed‐effect modeling showed that habitat covariates in nonhabitat sites were significantly lower than those in habitat sites (Table 4). For other significant variables such as dbhg10, Dbhg30, shrub diversity and tree diversity, the large amount of variability in mean response was due to the random effects of transects within site (Table 4) rather than random effect of site because tree species compositions were different for sites as elevation differs. The mean altitudinal range for sample habitat plots was 3,775 ± 234 m (mean ± standard deviation) with a range of 3,174 to 4,209 m and it was 3,505 ± 225 m for nonhabitat sites with a range of 3,252 to 3,978 m. However, the random effect of site and transect within site had a similar effect in variability of mean density of Dbh10 and slope. In an interesting manner, steeper slopes are preferred habitat over less steep ones.

**Table 4:** Comparison of least square means ± standard error for all measured habitat variables between habitat and nonhabitat sites based on expert view and variability contributed to the random effect associated with sites and with transect nested within site using multilevel mixed‐effect model

Variables	Expert view			Variability^a^	
	Habitat	Nonhabitat	*p*‐Value	*σ*_s_^2^ (%)	*σ*_ts_^2^ (%)
Altitude (m)	3,844 ± 81	3,452 ± 86	<0.001	71.1	26.4
CCPCT^b^ (%)	71.2 ± 2.0	21.5 ± 4.2	<0.001	—	—
Dbhg30 (stems/ha)	21 ± 4	4 ± 6	0.035	9.5	26.2
Dbhg10 cm (stems/ha)	188 ± 22	15 ± 57	<0.001	3.9	59.0
Dbh10 cm (stems/ha)	233 ± 42	17 ± 53	0.004	16.3	19.2
Dmht (m)	6.7 ± 0.6	5.6 ± 0.9	0.290	5.8	39.6
Shrub diversity^b^	2.4 ± 0.1	1.60 ± 0.2	<0.001	—	—
Slope (degree)	30.0 ± 3.0	14.7 ± 4.1	0.005	34.3	32.1
TPH (stems/ha)	443 ± 52	34 ± 74	<0.001	11.7	33.5
Tree diversity	1.5 ± 0.2	1.0 ± 0.2	0.089	20.4	32.8

*Notes*

Mean comparison of distance to water source was not conducted because the distance of water source was not measured for the nonhabitat sites.

^a^Variability contribution in mean estimates due to random effect of sites (*σ*_s_^2^) and random effect of transect within sites (*σ*_ts_^2^). ^b^Variability due to random effect of site was negative which indicated possibility of no site effect in the given mixed model structure, so contribution was not estimated.

#### 3.5.2 Multivariate analysis of habitat versus nonhabitat

**Figure 8:**
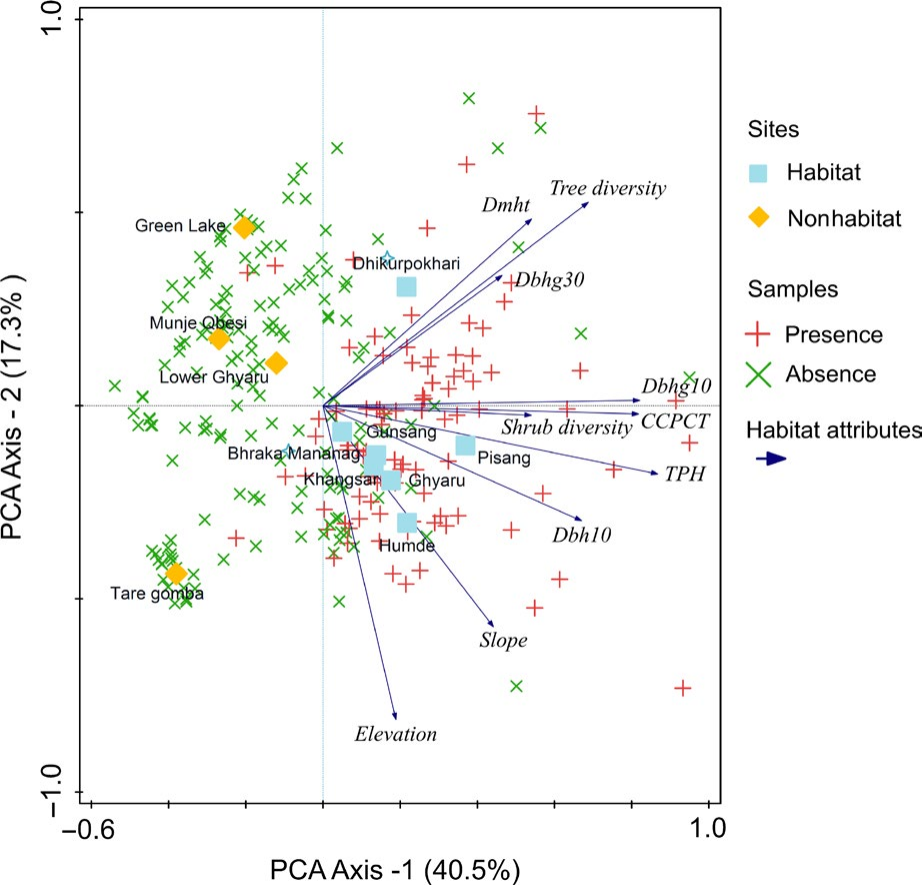
Bi‐plot of principal component analysis (PCA) showing relationship of habitat variables with habitat and nonhabitat sites and musk latrine absence and presence in Neshyang valley, Mustang, Nepal

The covariates of the habitat and nonhabitat sites were ordinated with principal component analysis (PCA; Figure 8). The first axis comprised 40.5% of all the variance and was highly attributed to TPH, CCPCT, Dbhg10 and Dbh10, shrub diversity, and Dmht. The second PCA axis comprised 17.3% and was mostly attributed to elevation and slope. The first four axes collectively comprised 78.6% of all the variance. The analysis showed that the response variables had a positive correlation with habitat sites (0.70) and latrine presence–absence samples (0.62), while the negative correlation with nonhabitat sites (−0.70) along the first axis. Similar to that, the habitat attributes had a strong positive correlation with habitat sites (0.89) and latrine presence–absence samples (0.79) while the strong negative correlation with nonhabitat sites (−0.89) along with the first axis. In contrast, the correlation of nonhabitat sites was positive but weak with both response variables (0.31) and habitat attributes (0.38) along the second axis. As well, the correlation of habitat site was −0.31 and −0.38 and of latrine presence–absence samples were −0.18 and −0.24 with both response variables and habitat attributes along the second axis respectively.

## 4 DISCUSSION

### 4.1 Latrine duration of use and location

We confirmed musk deer use of latrine sites for multiple years or at least more than 1 year based on no significant change in the number of latrines either fresh or old between 2 years of study. This result was similar to that found by Green (1986). Although musk deer use the same latrine site throughout all seasons of the year and for multiple years, the latrine sites could be used by more than one individual where territory overlaps (Green, 1987b). Because musk deer develop latrine sites by defecating at various locations, (e.g., under tree, under tree canopy and space under rock in the forest) the latrine sites also serve as a demarcation of territorial habitat use (Singh, Shrestha, Thapa, Saud, & Jiang, 2018). Among these locations, most commonly found location for a latrine was under tree location. One plausible explanation for that is shade under the canopy helps keep the dropping moistness and pungency for a longer period effectively maintaining chemical communication for a longer period. Selection of different locations as latrine sites along the landscape for multiple seasons and years helps to understand temporal and spatial habitat use of musk deer.

### 4.2 Relationship with topography and forest types

Some studies have shown that musk deer prefer southern aspects due to their warmer climatic conditions in the Himalaya (Green, 1985; Sathyakumar, 1994). However, in this study, high numbers of latrines were found in north, northeast and northwest aspects. Latrines were found on slopes ranging from 8° to 45° and were most abundant between 20° and 40°. Therefore, we suspect that preference of aspect and slope are rather a matter of habitat availability in relation to animal's ecological requirement than topographical attributes. Musk deer are generally found in the elevation range 2,000–5,000 m (Timmins & Duckworth, 2015). In our study, latrines were found highly concentrated between 3,650 and 4,000 m which means that musk deer are more frequent at higher elevation. This is because latrine site selection may also be influenced by a preference for birch and mixed fir forests, which occur at higher elevation range or for remoteness from human settlements. A prior study indicated that the elevation of 3,600–3,800 m was the most preferred elevation range in Manaslu Conservation Area (Subedi et al., 2012). Other studies from the western Himalaya region of India also showed a high frequency of latrines between 2,500 and 4,200 m (Ilyas, 2014) and a high probability of sighting musk deer above 3,000 m (Sathyakumar, 1994; Vinod & Sathyakumar, 1999). Musk deer are well adapted to high elevation habitats with low temperatures (Futuyma & Moreno, 1988). The hollow hair of musk deer is thick and provides adequate insulation to allow musk deer to remain at these elevations even during the winter (Green, 1985; Kattel, 1993).

Latrines were noted in both heterogeneous (mix of pine, fir, and birch or mix of any two species) and homogenous forests (single stand of either pine, fir or birch) located between 3,200 and 4,200 m, but birch and fir mixed forests were more strongly associated with latrine presence than pure fir and pine forests. Fir forest and birch forest are the most preferred habitats in Sagarmatha National Park (Aryal, Raubenheimer, Subedi, & Kattel, 2010), whereas Shrestha and Meng (2014) showed mixed forest and rhododendron forest is the most suitable habitat in the Gaurishankar Conservation Area, Nepal. In the central Himalayas of Nepal Khadka and James (2016) worked in smaller patches of pine and fir stands and found that only pine and fir stands were preferred habitat for musk deer. However, other studies in the Himalayan ranges of Nepal and India have reported that musk deer preferred a wide range of forest types found between 2,500 and 4,200 m (e.g., fir‐birch forests, mixed forests of birch, dwarf rhododendron scrub, birch‐rhododendron forests, fir and rhododendron, oak (*Quercus semecarpifolia*; Ilyas, 2014; Kattel, 1993; Shrestha & Meng, 2014). Therefore, forests at higher elevation in the Himalayas are highly essential for musk deer.

### 4.3 Microhabitat association

Stand‐level attributes, such as crown cover percentage, dominant tree height, shrub diversity and tree density have a significant influence on determining the presence of musk latrines. High total tree density (>350 stems/ha), small sized tree density (Dbh10) and crown cover 70%–90% were significantly correlated in choosing latrine sites. These stand characteristics of latrine sites help to retain latrine scents for longer time than the exposed sites and provide escape cover from predators. Because musk deer communicate through olfaction, scent retained in latrine and paste secretion on vegetation help to establish communication with other individuals (Green, 1985). As a result, most of the latrines were discovered under tree and canopy cover. Musk deer frequently refreshed established latrine sites under vegetation cover (tree and canopy) by leaving droppings rather than on latrine sites on space under rocks. Such latrine sites were mostly observed in mixed stand forest rather than pure stand forest having a higher stem density and vegetation cover.

Other studies confirm that musk deer presence and preference of latrine sites are significantly linked to vegetation density and the height of shrubs at the latrine site (Ilyas, 2014), as well as increased crown cover (>42%; Khadka & James, 2016). Such site characteristics also help them hind within vegetation cover throughout the daylight where they remain to avoid detection by predators. Musk deer are concentrate feeders which helps them cope with a poor quality diet and consequently depend on shrub species for a major portion of their diet (Green, 1987a). Musk deer mostly feed during dawn, dusk and at night and avoid open pasture away from forest for browsing (Green, 1985; Kattel, 1993). Livestock use in the study area is intermittent and musk deer and cattle have different dietary consumption preferences (Khadka, Singh, Magar, & James, 2017; Syed & Ilyas, 2016). However, the impact of livestock grazing cannot be ignored because overgrazing leads to forest fragmentation and depletion of palatable browse species (Bakker, 1998; Mayer, Kaufmann, Vorhauser, & Erschbamer, 2009).

PCA analysis also clearly showed the distinction between habitat used for latrines and nonhabitat not used by musk deer. The first axis of PCA distinguishing between habitat and nonhabitat sites highly correlates to habitat variables indicating that habitat variables played an important role in characterizing musk habitats versus nonhabitats and latrine absence versus presence. The comparative results between latrine presence and absence sites in the Neshyang Valley revealed similar associations of stand attributes with latrine site use. A comparison between forests where musk deer were never recorded and the habitat of the musk deer also verified that crown cover, tree height, tree diversity, diameter of trees, and shrub diversity plays an important role for musk deer presence in a particular site and discriminate other topographical attributes. The association of musk deer latrines with stands that have greater tree height and varying diameter (Table 2) strongly indicate that musk deer prefer dense stands. Both linear models and ordination method indicate that stand attributes such as crown cover percentage, dominant height, tree diameter, tree diversity and shrub diversity are important attributes for determining the presence of musk deer in forest located between 2,500 and 4,300 m.

## 5 CONSERVATION RECOMMENDATIONS

Musk deer are highly associated with dense and undisturbed forests of the higher Himalayas because of their unique behavior and timid nature. Habitat degradations cause forest fragmentation, depletion of tree and shrub cover, and reduction of floral diversity. As a result, escape cover and food resources available to the musk deer can be reduced or eliminated. For the first time, this study has given quantitative relationship of musk deer with habitat parameters such as tree crown cover, tree height, tree diversity, the diameter of trees, and shrub diversity in the forest between the altitude of 2,500 m and 4,300 m. The magnitude of relationships can be used as a threshold while managing habitat of musk deer in Nepal and other courtiers. Therefore, these findings of this study can be incorporated into present management practices in the study area which can then serve as a template for musk deer's habitat management in other areas in the Himalayas. This research has mainly emphasized latrines of musk deer as mean to understand the presence of deer and their abundance in a particular habitat. Latrines are located spatially in the forest and musk deer use them temporarily for many years. Therefore, the location of latrines can be referred to plan systematic and long‐term forest patrolling to curtail poaching which has been considered as a serious threat to musk deer because of demand of musk in various countries around the world. However, patrolling should be conducted randomly in different habitats so that musk deer would not be dissuaded from using latrine site. Indeed, we hope that this study will provide baseline information used to prepare species conservation action plans. Using the approach based on latrine sites, we hope that future studies can be designed to investigate the response of musk deer to livestock grazing and human disturbances, latrine site territorial claims by different sexes and individuals, predator escape behavior, and territorial behavior. We suggest that advanced technology such as molecular genetic analysis, camera trapping, and use of GPS collars would be useful in this endeavor.

## CONFLICT OF INTEREST

None declared.

## AUTHORS CONTRIBUTION

PBS, PS, and JZ were involved with the concept and design. PBS collected the samples; PS and PBS conducted the analysis. PBS led the writing and all authors were involved in interpretation of the results and in the writing process. All authors approved the final draft of the manuscript.

## DATA ACCESSIBILITY

Biophysical data are available from Dryad Digital Repository: https://doi.org/10.5061/dryad.v59dh02.
